# Enhancing methane yield and microbial resilience in olive pomace anaerobic digestion via co-digestion with pig manure

**DOI:** 10.1186/s13068-025-02711-9

**Published:** 2025-11-18

**Authors:** Sandra Correa, Mercedes Llamas, Fabiana Passos, Soraya Zahedi, José Manuel Espinosa, Fernando G. Fermoso, Ivet Ferrer

**Affiliations:** 1 GEMMA - Environmental Engineering and Microbiology Research Group, Department of Civil and Environmental Engineering, Universitat Politècnica de Catalunya - BarcelonaTech, c/ Jordi Girona 1-3, Building D1, 08034 Barcelona, Spain; 2 Instituto de la Grasa, Spanish National Research Council (CSIC), Campus Universitario Pablo de Olavide, Ed. 46, Ctra. de Utrera, km. 1, 41013 Seville, Spain; 3 Circular Economy Process Design, Repsol Technology lab, 28935 Móstoles, Madrid Spain

**Keywords:** Agro-industrial waste, Bioenergy, Biogas, Circular bioeconomy, Energy transition, Olive oil, Renewable energy

## Abstract

**Background:**

Intensive agricultural practices are increasing the generation of by-products and wastes, which require appropriate management strategies to prevent environmental pollution and recover valuable resources. Waste-to-energy technologies, such as anaerobic digestion, are gaining attention for integrating local feedstocks to produce biofuels and biofertilisers, contributing to closed nutrient cycles. In the Mediterranean region, olive pomace is very abundant, but its intrinsic characteristics hinder the production of biogas via anaerobic digestion. Concurrently, the direct application of untreated pig manure on agricultural land continues to pose significant environmental risks.

**Results:**

This study assessed the biomethane potential of olive pomace and pig manure, along with microbial population dynamics during the transition from mono- to co-digestion. Mono-digestion of olive pomace led to complete process inhibition, while co-digestion with pig manure increased methane yield more than fivefold (from 53 to 283 mL CH_4_ g^−1^ VS). Co-digestion also enhanced the microbial diversity, improving the ecosystem resilience and metabolic versatility. A notable increase in the relative abundance of methanogenic archaea, particularly *Methanosarcina*, was observed*.* An energy assessment indicated that a full-scale plant co-digesting olive pomace and pig manure could not only operate without external energy consumption, but also produce excess electricity (577 MWh y^−1^) and heat (1074 MWh y^−1^).

**Conclusions:**

These findings demonstrate that co-digestion can overcome the limitations of olive pomace mono-digestion, enabling the effective treatment of two challenging agro-industrial by-products. This approach aligns with circular bioeconomy principles and supports the decarbonisation of the olive oil and pig farming sectors, contributing to the energy transition. The presented approach serves as a baseline scenario, and further research should focus on recovering high-value bioproducts and advancing towards integrated biorefinery systems in rural areas.

**Graphical abstract:**

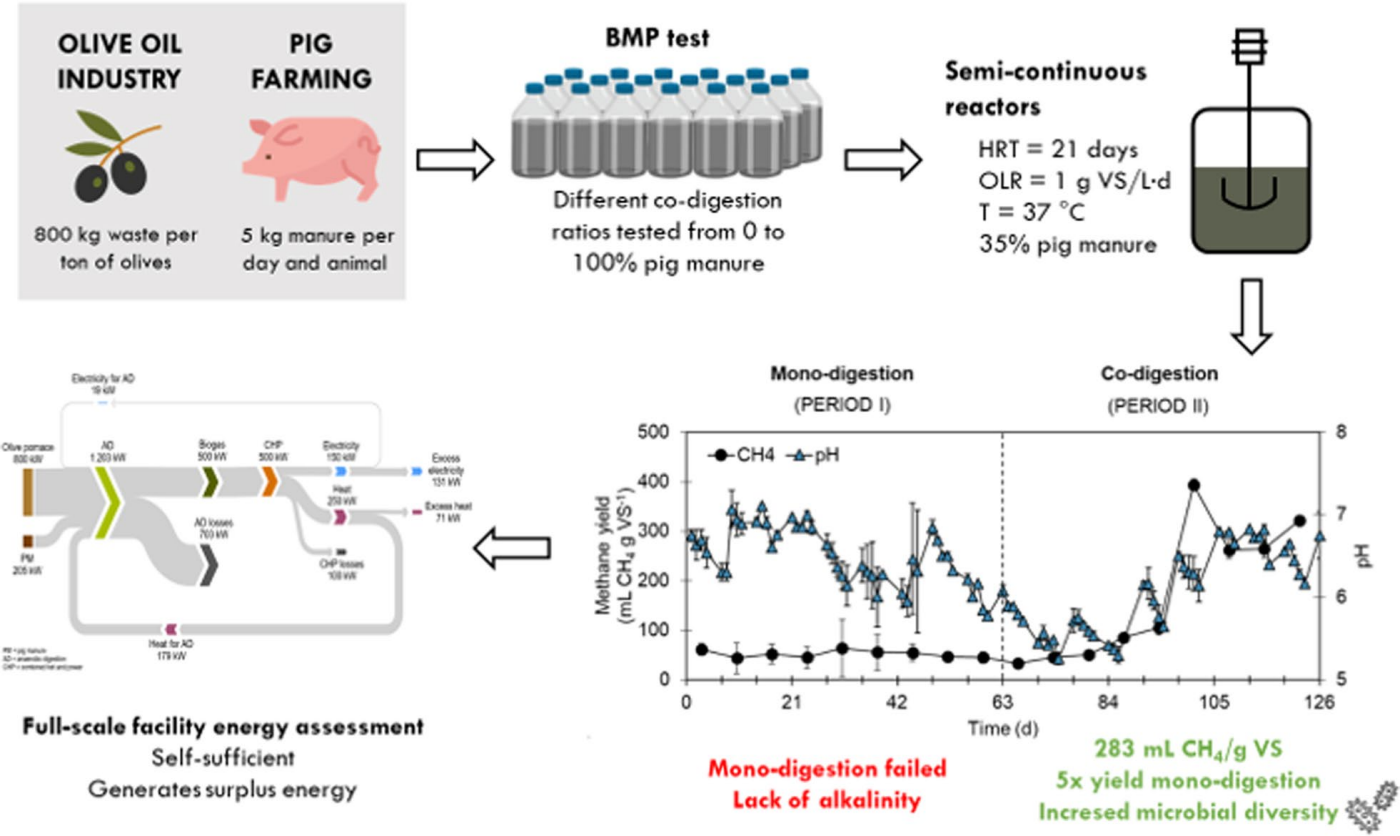

**Supplementary Information:**

The online version contains supplementary material available at 10.1186/s13068-025-02711-9.

## Background

The valorisation of agro-industrial residues through the production of bioenergy and bioproducts is increasingly recognised as a key strategy for advancing the circular bioeconomy and reducing the environmental impacts associated with conventional waste management [1]. According to recent estimates, agro-industrial activities contribute significantly to organic waste streams, many of which remain underutilised [2]. In Europe, efforts are focused on minimising organic waste generation with clear waste hierarchy guidelines that prioritise the recovery of energy and materials over landfilling [3]. Among the available waste-to-energy technologies, anaerobic digestion stands out as a mature and efficient biological process that converts organic matter into biogas and a stabilised digestate that can be used as a biofertiliser. Biogas is recognised as a strategic renewable energy carrier that can contribute not only to the decarbonisation of the sector, but also to improve energy security and nutrient recycling. EU biomethane targets for 2030 will require a fivefold production increase to reach 35 bcm annually, evidencing the need for further research and development [4].

The olive oil industry, a cornerstone of the Mediterranean agri-food sector, generates large volumes of by-products and residues, particularly olive pomace. This by-product is primarily generated by the two-phase olive oil extraction system, which is extensively used in Spain and other Mediterranean countries due to its low water requirement as compared to the three-phase extraction system [5]. Only in Spain, the largest olive oil producer in the world, over 5 million tonnes of olive pomace are generated every year [6]. Olive pomace is characterised by high humidity and low pH [7], and despite the substantial organic content, its lignocellulosic nature and high concentration of phenolic compounds and other aromatic molecules can inhibit anaerobic microbial activity [8, 9]. Indeed, the stable operation of these anaerobic reactors would require low organic loading rates (OLR) and long hydraulic retention times (HRT ~ 230 d) [9], thereby leading to large reactor volumes and increasing capital and operational costs. As a result, the mono-digestion of olive pomace remains technically and economically challenging at full scale.

Co-digestion strategies, particularly with nitrogen-rich substrates, offer a potential pathway to overcome these limitations. These co-substrates can help balance physiochemical properties, dilute toxic compounds, and enhance microbial activity while promoting regional co-treatment of agro-industrial wastes [10]. The selection of a suitable co-substrate depends on multiple factors, such as location, production volume or available technology. The co-digestion of olive pomace has previously been studied using microalgae [11], animal slurries [9, 12, 13], sewage sludge [14], food waste [15], and other agro-industrial by-products [16]. However, reported methane yields vary substantially, ranging from about 100 to nearly 600 mL CH_4_ g^−1^ volatile solids (VS). Pig manure and slurries, which are widely available often near olive mills, have shown great potential as a co-substrate [13]. In Spain in particular, this residue tends to be inadequately managed, posing a significant environmental hazard and contributing to freshwater pollution due to its nitrate content [17]. Although anaerobic digestion of animal manure or slurry is a widespread technology, problems such as carbon-to-nitrogen (C/N) imbalances and ammonia inhibition are often reported [18]. Co-digesting it with carbon-rich residues like olive pomace may create a synergistic effect, improving biogas yields and overall process stability.

Despite these promising synergies, current literature on the co-digestion of olive pomace is limited, especially at bench or pilot scale [9, 13–16, 19]. Most studies have focused on batch trials, with very limited insights into microbial dynamics, long-term reactor performance, or energetic feasibility in real-world conditions. Therefore, this study investigates the mono- and co-digestion of olive pomace and pig manure as a strategy to valorise two abundant and polluting agro-industrial by-products. To this end, different co-digestion ratios of olive pomace and pig manure were initially tested in batch tests. Subsequently, the mono-digestion and co-digestion were studied in bench-scale semi-continuous reactors, monitoring reactor process performance and microbial population dynamics and evolution. Finally, an energy balance was conducted to assess the feasibility of implementing such a co-digestion system at full scale, within the framework of a sustainable and decentralised circular bioeconomy.

## Materials and methods

### Substrates and inoculum

Olive pomace was obtained from a large-scale storing and drying facility located in Marchena (Seville, Spain). The collected pomace corresponded to the residual stream from two-phase olive mills. Pig manure used as co-substrate was collected from a local farm in Seville (Spain). The inoculum employed to perform the batch tests was digested sludge from a full-scale mesophilic anaerobic digester at an urban wastewater treatment plant located in Barcelona (Spain), while semi-continuous reactors were inoculated with mesophilic digestate from semi-continuous reactors fed with thermally pre-treated olive pomace. Both substrates were frozen ( − 20 °C) until use, while inocula were used fresh. Their physicochemical characterisation is shown in Table 1.

**Table 1:** Physicochemical characterisation of two-phase olive pomace, pig manure and inocula used in batch tests (digested sludge from a wastewater treatment plant) and semi-continuous tests (adapted sludge from bench-scale reactors)

Parameter	Olive pomace	Pig manure	Inoculum (batch test)	Inoculum (semi-continuous reactors)
pH	4.58 ± 0.10	6.53 ± 0.12	7.46 ± 0.04	6.75 ± 0.07
TA (g CaCO_3_ kg^−1^)	*n*/d	25.79 ± 1.01	1.34 ± 0.01	3.36 ± 0.09
EC (mS cm^−1^)	1.41 ± 0.03	1.29 ± 0.02	–	1.82 ± 0.01
TS (g kg^−1^)	252.60 ± 3.00	374.44 ± 4.65	18.63 ± 0.06	27.39 ± 0.71
VS (g kg^−1^)	236.85 ± 3.12	218.43 ± 10.01	11.52 ± 0.04	22.13 ± 0.77
VS/TS	0.94 ± 0.03	0.58 ± 0.06	0.45 ± 0.06	0.81 ± 0.01
COD (g O_2_ kg^−1^)	351.17 ± 3.55	263.62 ± 4.58	15.90 ± 0.94	–
Soluble COD (g O_2_ kg^−1^)	135.35 ± 1.75	51.96 ± 4.88	0.26 ± 0.05	10.07 ± 0.12
C/N	34.59 ± 1.18	16.89 ± 1.17	–	30.61 ± 1.97
N–NH_4_^+^ (mg N kg^−1^)	147.93 ± 7.85	832.85 ± 24.98	302.09 ± 4.44	–
Total phenols (g GAE kg^−1^)	10.18 ± 0.35	–	–	1.55 ± 0.14
Soluble phenols (g GAE kg^−1^)	4.82 ± 0.58	–	–	0.47 ± 0.01
Dihydroxyphenylglycol (mg kg^−1^)	295.80 ± 5.79	–	–	–
Hydroxityrosol (mg kg^−1^)	2749.65 ± 64.75	–	–	–
Tyrosol (mg kg^−1^)	798.63 ± 10.64	–	–	–

Substrates were kept frozen (-20 °C), while inocula were used immediately after collection to avoid degradation. Mean and standard deviation (*n* = 3)

TA: total alkalinity; EC: electrical conductivity; TS: total solids; VS: volatile solids; COD: chemical oxygen demand; C/N: carbon-to-nitrogen ratio; N–NH_4_^+^: ammonium nitrogen; GAE: gallic acid equivalent; *n*/d: not detected

### Biochemical methane potential tests

The co-digestion of olive pomace and pig manure was initially assessed by means of biochemical methane potential (BMP) tests at different ratios (i.e., 10%, 20%, 35%, 50%, 65% and 80% of pig manure on a VS basis), following the protocol described by Ruales et al. [20], and based on standard guidelines [21]. The mono-digestion of olive pomace and pig manure (0% and 100% pig manure, respectively) was used as a control. A blank (only inoculum) and a positive control (inoculum and cellulose) were also included in the assay. Tests were performed using 160 mL glass bottles (120 mL of useful volume and 40 mL of headspace) with an initial concentration of organic matter of 16.7 g VS L^−1^ and an inoculum-to-substrate ratio of 2 (on a VS basis). Bottles were flushed with helium at the beginning of the assay and sealed with septum caps to ensure anaerobic conditions. The experiment was conducted under mesophilic conditions (37 °C) using a model I10-OE incubation chamber (OVAN, Barcelona, Spain) provided with mechanical agitation (75 rpm). Biogas production was monitored periodically by registering the pressure generated inside the bottles using a GMH 5550 handheld device (GHM Messtechnik GmbH, Greisinger, Germany) and samples of the headspace were analysed weekly to determine the composition of the biogas. All conditions were studied in triplicate and BMP tests were monitored until biogas production ceased. Results were expressed as average values of accumulated methane production under normal conditions by subtracting the blank trial and dividing by the initial VS content of the substrate(s) in each trial (mL CH_4_ g^−1^ VS).

According to the literature, first-order kinetics were used to model olive pomace degradation, given that hydrolysis is the limiting step of the anaerobic bioconversion of particulate organic matter [22]. Therefore, experimental data were fit to the following exponential Eq. (1), where *B* (mL CH_4_ g^−1^ VS) is the methane yield at time *t* (d), *B*_*0*_ (mL CH_4_ g^−1^ VS) is the maximum methane yield and *k* (d^−1^) is the first-order kinetics (i.e., hydrolysis) constant:

1 $$B={B}_{0}(1-\text{exp}\left(-k t\right))$$

Least-squares fitting was performed in *Microsoft Excel* software and the goodness of the model fitting was evaluated by the coefficient of determination (R^2^) and the standard error of the estimate (SEE). Experimental methane yields and model parameters were analysed using one-way analysis of variance (ANOVA), followed by Tukey’s post hoc test to assess significant differences among co-digestion ratios (*α* = 0.05).

### Semi-continuous reactors

The anaerobic mono-digestion of olive pomace and its co-digestion with pig manure were studied in semi-continuous stirred tank reactors (CSTR) with a total volume of 2 L and a working volume of 1.7 L. Experiments were performed in duplicate using a thermostatic chamber to ensure mesophilic conditions (37 ± 1 °C), a HRT of 21 d and an OLR of 1 g VS L_R_^−1^ d^−1^. Reactors were operated for 126 days divided into two periods, namely, Period 1 (from days 1 to 63), when the mono-digestion of olive pomace was studied, and Period 2 (from day 64 onwards), when the co-digestion with pig manure (35% on a VS basis) was evaluated.

Daily methane production was measured by water displacement using Mariotte’s bottles containing water at 25 °C. 250 mL sealed bubblers placed between the reactor and the Mariotte’s bottles, filled with a 3N NaOH solution, were used to remove CO_2_ from the biogas. In addition, liquid traps were placed before the bubblers to prevent NaOH reflux to the reactors due to pressure imbalances in the system.

The methane yield, pH and soluble chemical oxygen demand (COD) were monitored daily. The concentration of volatile fatty acids (VFA) was measured three times per week. Total solids (TS), VS, phenols and total alkalinity (TA) were analysed twice per week. Moreover, seven samples were extracted directly from each reactor on days 0, 21, 42, 63, 84, 105 and 126 to monitor the microbial community and stored at 80 °C until further processing.

Process performance was evaluated by the anaerobic biodegradability (BD), as described in the following equation:

2 $$BD (\%)=\frac{mL {CH}_{4 }/g {VS}_{in}}{\left(350 mL {CH}_{4 }/g COD\right)\left(1.48 \%olive pomace+1.21 \%pig manure\right) } 100$$

where 1.48 and 1.21 correspond to the COD-to-VS ratio for olive pomace and pig manure, respectively.

### Analytical methods

The pH and electrical conductivity (EC) were measured using a pH meter (MM150 senION + , HACH, USA). TA was determined by pH titration to 4.5 using an automatic titration system (HI901, Hanna Instruments, USA). TS and VS were determined according to the APHA procedure 2540G [23]. C/N ratio was calculated from an elemental microanalysis performed using a LECO elemental analyser (CH828, LECO, USA). Ammonium nitrogen (N–NH_4_^+^) was evaluated by distillation and subsequent titration of the distillate with sulphuric acid [23]. COD was determined following the open reflux method proposed for substrates with high suspended solid content [24]. Total phenols were quantified after extraction with a methanol/water solution (80:20) and filtration (disk filter 0.45 μm), according to the Folin–Ciocalteu method [25], and measured with a spectrophotometer (iMark Microplate Reader, Biorad, USA).

Soluble COD, VFA and soluble phenols were determined from the soluble fraction, obtained after centrifugation at 4700 rpm for 20 min (Centrifuge Digtor 22, Ortoalresa, Spain) and filtration (disk filter 0.45 μm). Soluble COD was determined after closed digestion of the samples by the colorimetric standard method 5220D [23]. The concentration of individual VFA (acetic, propionic, butyric, iso-butyric, valeric and iso-valeric), was measured using a gas chromatograph (GC-2014, Shimadzu, Japan) equipped with a 30 m × 0.25 mm Stabilwax fused silica capillary column and a flame ionisation detector. The temperature of the oven was increased from 100 to 150 °C using a heating rate of 4 °C min^−1^. Soluble phenols were determined using the aforementioned Folin–Ciocalteu method on the soluble fraction. Individual soluble phenols were determined in a high-performance liquid chromatography (HPLC) system (Beckman Coulter, USA) equipped with a System Gold 168 detector, a solvent module 126 and a stainless-steel Merck Superspher RP-18 (250 × 4 mm) column. Acidified water (2.5% formic acid) and acidified methanol (2.5% formic acid) were used as mobile phases. The system was fitted with a 20 μL sample injection loop and the detection was performed at 280, 350 and 510 nm.

DNA extraction and Library Preparation was performed as described by Trujillo-Reyes et al. [26]. For total DNA extraction, 2 mL of sample were centrifuged at 13000 rpm and the pellet was processed using FastDNATM SPIN Kit for Soil (MP Biomedicals). Two biological samples were used on each datapoint. Data analysis was performed following Cubero-Cardoso et al.[27]. Briefly, the V4 region of the 16S rRNA gene was amplified using barcoded primers via one-step PCR. The PCR reaction included 10–20 ng of DNA and standard reagents, and amplification was performed using an Alpha cycler (PCRmax, Biorad, UK). PCR products were purified, quantified, pooled in equimolar concentrations, and sequenced on the Illumina NovaSeq 6000 platform (Eurofins Genomics, Germany). Raw reads were processed using NG-Tax with SILVA 138.1 as the reference database. Bacterial composition and alpha and beta diversity were calculated using Kraken software suite [28]. Abundances were calculated using the Bracken method [29].

### Energy assessment

**Fig. 1:**
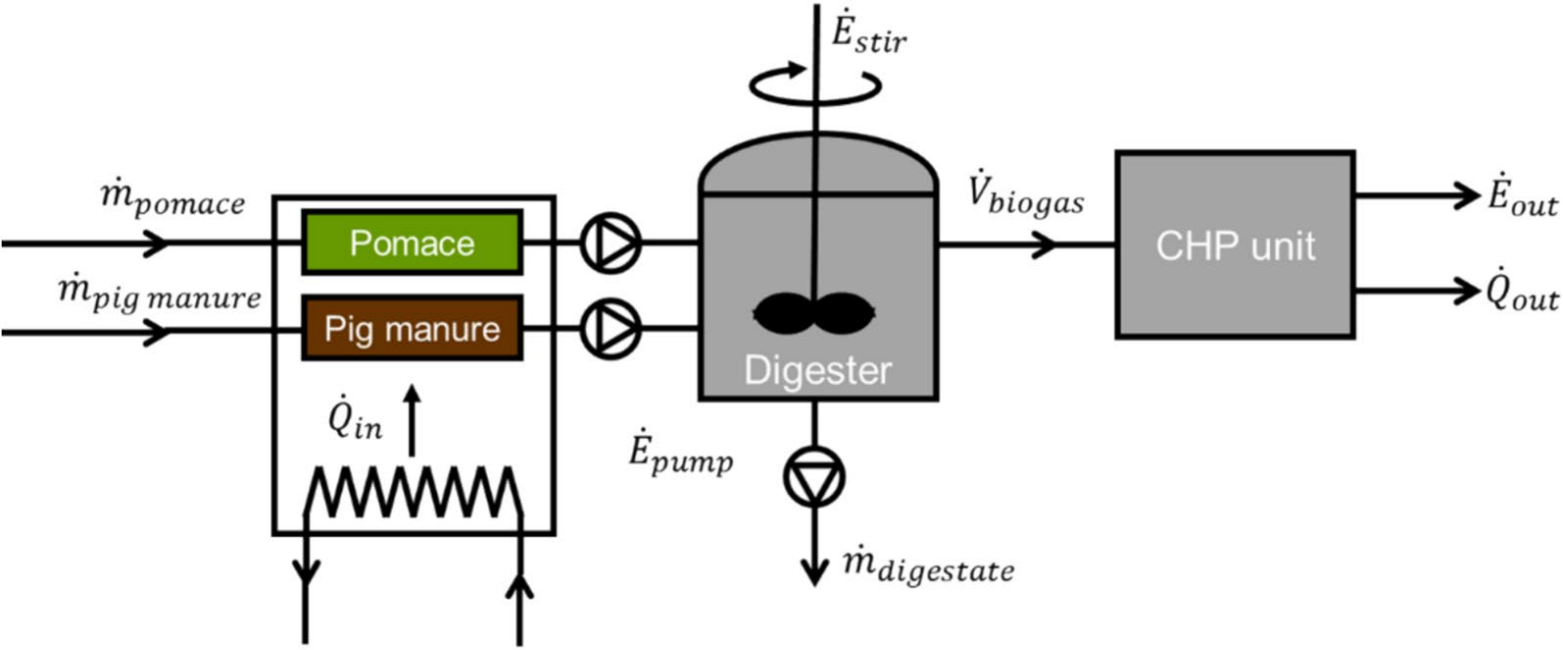
Layout of the proposed facility with an anaerobic digester and a combined heat and power (CHP) unit, showing energy and mass flows (ṁ = mass flow rate; V̇ = volumetric flow rate; Q̇ = heat flow rate; Ė = electricity flow rate). The plant was designed to treat 4000 t y^−1^ of olive pomace and 2200 t y^−1^ of pig manure; the nominal capacity of the CHP unit is 150 kW_e_ (250 kW_th_), operating 8000 h y^−1^.

Experimental data were used to perform an energy balance for a potential industrial-scale anaerobic digestion plant for the co-digestion of olive pomace and pig manure in a Mediterranean region. In particular, Catalonia (northeast Spain) was identified as a hot spot for the implementation of this technology based on the annual generation and geographical distribution of olive pomace and pig manure. The plant was designed to treat 4000 t y^−1^ of olive pomace (equivalent to a medium-sized olive mill generating ~ 1000 t y^−1^ of olive oil), and the pig manure from a pig farm with 1200 heads (generating ~ 2200 t y^−1^ of pig manure). The facility included a combined heat and power (CHP) unit fuelled by the biogas generated in the digester (Fig. 1) and working 8000 h y^−1^ [30]. Based on the experimental methane yield obtained in this study, the calculated nominal capacity for the CHP unit was 150 kW_e_ (250 kW_th_).

Energy and mass balances were carried out to assess the self-sustainability of the proposed full-scale facility and the potential production of excess energy. Energy balances considered the power requirements of the proposed system (both thermal and electric) and the energy produced. The latter was calculated from the average experimental methane yield obtained upon steady-state operation of the co-digestion period (from days 105 to 126). Heat requirements were calculated as the thermal energy needed to raise the temperature of the substrates from 20 °C (average annual temperature in southern Catalonia) to 37 °C, and an additional 10% to compensate for the thermal losses of the digester [5]. Electricity consumption of the plant corresponded to pumping (1800 kJ m^−3^) and digester stirring (300 kJ m^−3^ d^−1^) [5]. The biogas was fed to a small CHP unit. Electric and thermal efficiencies were assumed to be 30% and 50%, respectively, where the CHP electricity self-consumption was already considered [31].

## Results and discussion

### Biochemical methane potential tests

**Fig. 2:**
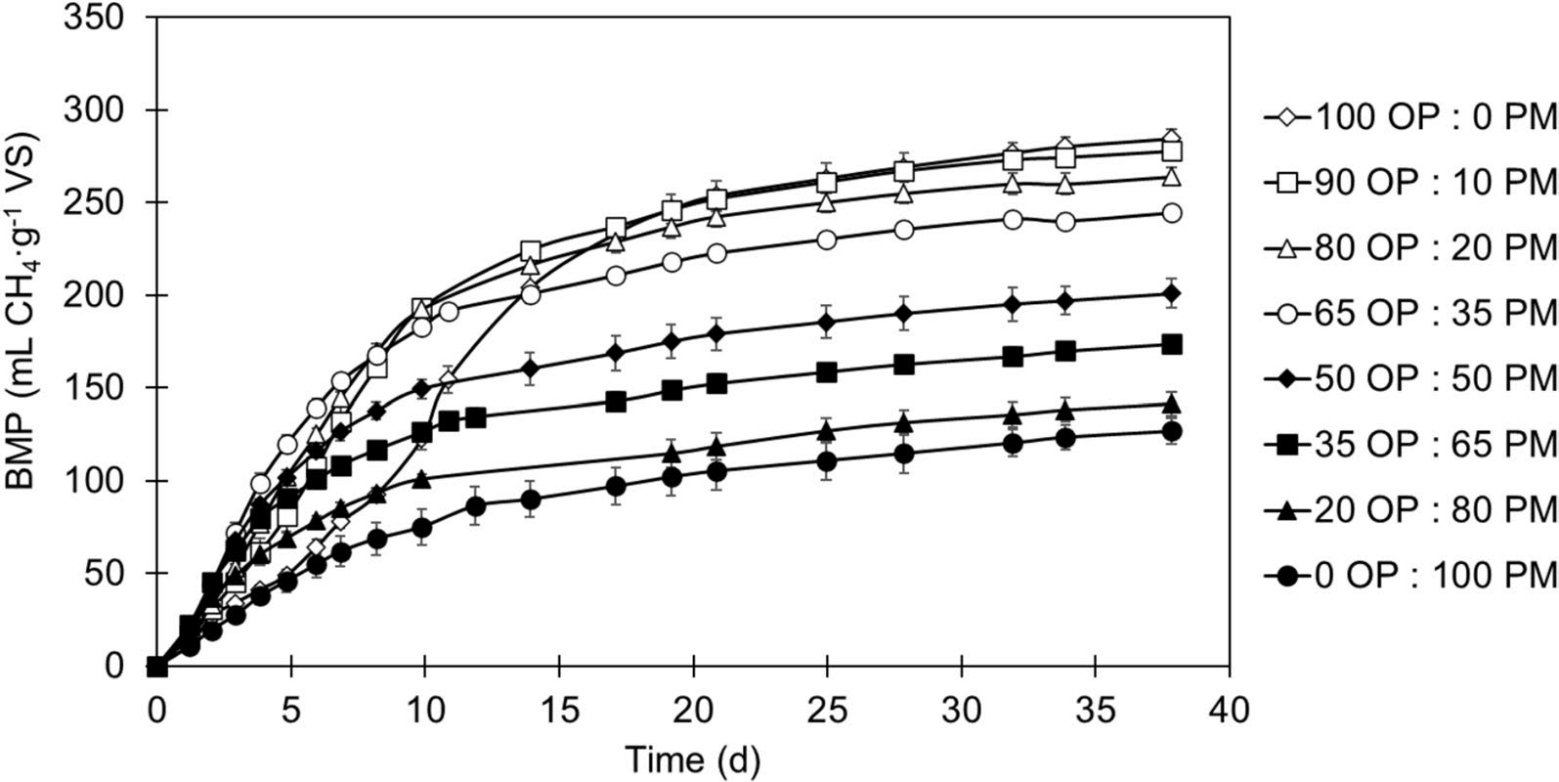
Biochemical methane potential (BMP) expressed as accumulated methane volume per mass of volatile solids (VS) added, for the mono-digestion and co-digestion of olive pomace (OP) and pig manure (PM) at different ratios on a VS basis. Results are given as mean and standard deviation (error bars) (*n* = 3). Experiments were run under mesophilic conditions (37 °C) and constant agitation (75 rpm). Inoculum was digested sludge from a wastewater treatment plant and the inoculum-to-substrate ratio was fixed at 2 (VS basis). Only distilled water was added to dilute the substrates and reach a useful volume of 120 mL

The methane yield of olive pomace and pig manure upon mono- and co-digestion was initially studied in BMP tests (Fig. 2). The maximum methane yield was achieved with 100% olive pomace (284.4 mL CH_4_ g^−1^ VS) and decreased as the pig manure concentration increased, reaching 126.8 mL CH_4_ g^−1^ VS (100% pig manure). In fact, the anaerobic biodegradability of pig manure (27%) was half that of olive pomace (54%). A higher methane yield (321 mL CH_4_ g^−1^ VS) was reported for the mono-digestion of olive pomace upon trace element solutions addition [32]. In this manner, it is also possible that olive pomace anaerobic biodegradability was enhanced due to the input of micronutrients and trace elements from the inoculum.

Synergistic and antagonistic effects related to the co-digestion were evaluated by comparing empirical and theoretical values for each co-digestion ratio. Theoretical values (CH_4,th_) were calculated according to Eq. (3), where 284.4 and 126.8 are the experimental methane yields (mL CH_4_ g^−1^ VS) obtained for the mono-digestion of olive pomace (100% olive pomace) and pig manure (100% pig manure), respectively, and % olive pomace and % pig manure correspond to the percentages of olive pomace and pig manure in each co-digestion mixture:

3 $${CH}_{4, th}=\% olive pomace 284.4+\% pig manure 126.8$$

**Fig. 3:**
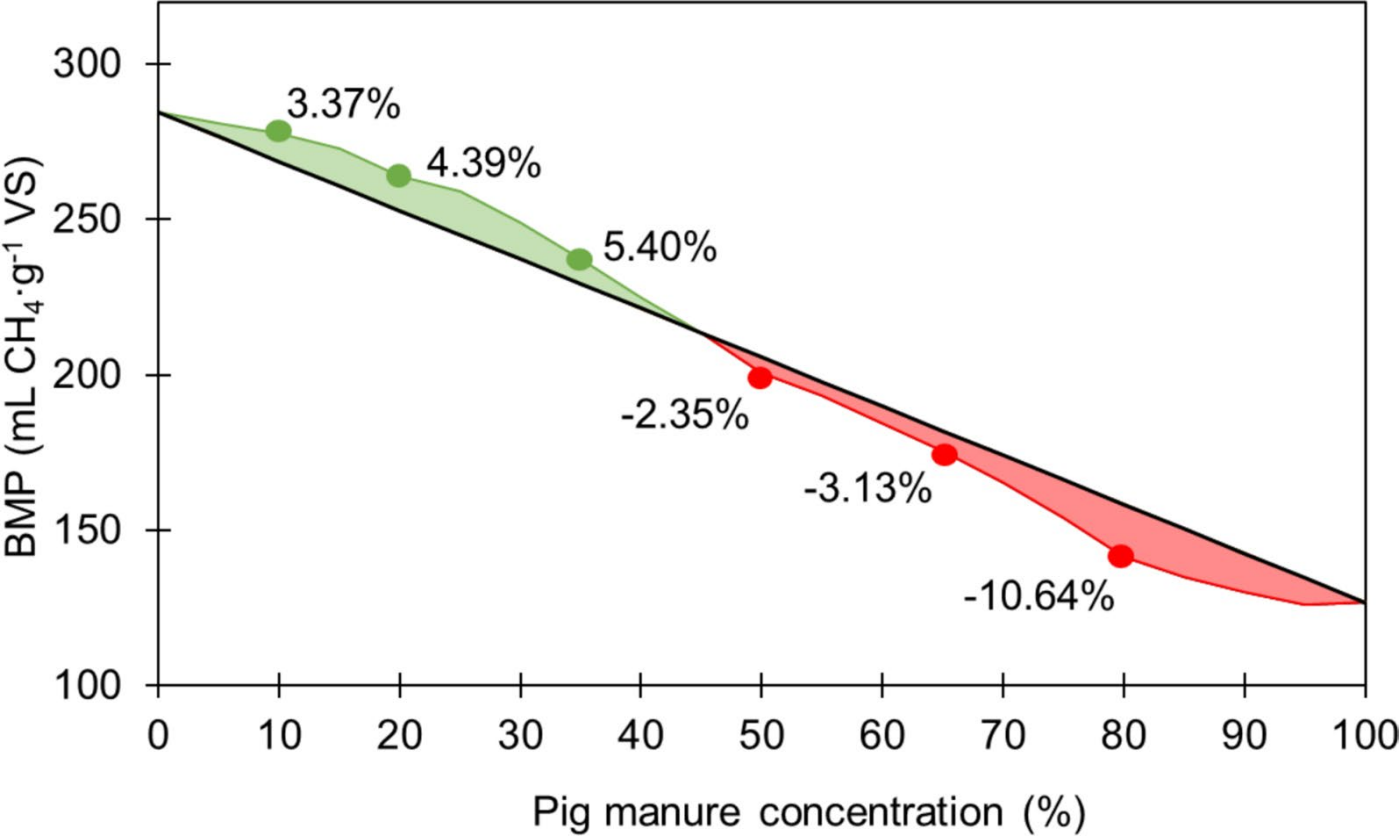
Differences between the experimental and calculated biochemical methane potential (BMP), expressed as increase or decrease (%), for different co-digestion ratios (10%, 20%, 35%, 50%, 65% and 80% pig manure on a volatile solids (VS) basis). The calculated BMP was obtained by linear interpolation between the experimental mono-digestion values for olive pomace (284.4 mL CH_4_ g^−1^ VS) and pig manure (126.8 mL CH_4_ g^−1^ VS)

Figure 3 shows that the co-digestion mixture containing 65% olive pomace and 35% pig manure had the greatest synergistic effect among them all. The experimental methane yield for this mixture was 5.4% higher than the theoretical value, i.e., 244.4 vs. 231.9 mL CH_4_ g^−1^ VS, respectively. Similar synergistic effects have been reported for the co-digestion of olive pomace with other nitrogen-rich substrates, such as cattle manure or microalgae [11, 12]. On the other hand, antagonistic effects were detected when the co-digestion ratio exceeded 35% of pig manure, as the experimental values were lower than the calculated ones. This can be attributed to a preference for pig manure conversion due to more readily available organic matter, in contrast to olive pomace that presented an initial lag phase upon mono-digestion [33]. Conversely, Al Afif et al. [34] observed antagonistic effects for both high and low concentrations of olive pomace when co-digested with cattle manure, but synergistic effects using a 50:50 co-digestion ratio.

The results of the model fitting, together with the experimental methane yield and anaerobic biodegradability are shown in Table 2. *R*^*2*^ values were higher than 0.99 in all cases, except for the trials with 100% olive pomace and 10% pig manure. Likewise, mixtures with a higher content of pig manure showed lower SEE values, which indicates a better fitting of experimental values to the proposed model. A significant improvement (*P* < 0.05) in the hydrolysis constant was observed for mixtures containing 35% or more pig manure; however, this resulted in a decrease in maximum methane production. In fact, the mono-digestion of olive pomace showed the poorest model fitting due to an initial lag phase, which could be attributed to the high presence of complex yet biodegradable compounds in olive pomace that prolong the hydrolysis stage. It should be noticed that the high proportion of inoculum in BMP tests provided alkalinity and increased the buffer capacity in comparison with olive pomace, favouring the slow conversion of intermediate compounds into biogas that otherwise may remain undigested [35]. The highest *k* value was obtained for 65% pig manure co-digestion ratio, i.e., 0.153 d^−1^. Slightly lower values were obtained for 35%, 50% and 80% of pig manure, with differences of less than 10%, i.e., 0.138 d^−1^,0.147 d^−1^ and 0.145 d^−1^, respectively. The time required to produce 80% of the methane (t_80_) was also calculated for each condition (Table 2). *t*_*80*_ values fluctuated among co-digestion ratios. Indeed, mixtures containing 35%, 50%, 65% and 80% of pig manure needed around 11 d to produce 80% of the maximum methane yield. Despite the higher methane yield, 100% olive pomace required threefold the time to reach the same point. Indeed, long lag phases have been reported for phenol-rich substrates like olive pomace, leading to longer HRT to achieve high bioconversion rates [36].

**Table 2:** Kinetic parameters obtained by fitting the first-order kinetics model ${\varvec{B}}={{\varvec{B}}}_{0}(1-\mathbf{exp}\left(-{\varvec{k}}\boldsymbol{ }{\varvec{t}}\right))$ to experimental data obtained in biochemical methane potential (BMP) tests with different olive pomace and pig manure co-digestion ratios

Pig manure (% VS)	CH_4,exp_ (mL CH_4_ g^−1^ VS)	BD (%)	B_0_ (mL CH_4_ g^−1^ VS)	k (d^−1^)	R^2^	SEE	t_80_ (d)
0	284.4^a^	54.3%	357.1	0.050^e^	0.983	20.04	31.89^a^
10	277.7^ab^	53.6%	293.8	0.092^d^	0.990	15.18	17.43^b^
20	264.0^b^	51.2%	271.0	0.110^c^	0.994	10.84	14.64^c^
35	244.4^c^	48.2%	241.0	0.138^b^	0.997	6.41	11.70^d^
50	200.8^d^	40.3%	193.2	0.147^b^	0.996	5.07	10.98^d^
65	173.7^e^	35.5%	164.0	0.153^a^	0.995	5.09	10.52^d^
80	141.5^f^	29.2%	135.0	0.145^b^	0.997	3.66	11.08^d^
100	126.8^f^	26.8%	127.6	0.088^d^	0.998	2.28	18.20^b^

Least-squares fitting was performed in *Microsoft Excel* software and results are given as the mean value (*n* = 3)

VS: volatile solids; CH_4,exp_: experimental methane yield; BD: anaerobic biodegradability; B_0_: maximum methane production; k: hydrolysis rate; R^2^: coefficient of determination; SEE: standard error of estimate; t_80:_ time to reach 80% of the methane yield

Different letters indicate significant differences (*P* < 0.05) according to Tukey’s post hoc test

According to the methane yield, synergistic effects and kinetic parameters, a co-digestion ratio of 35% pig manure appeared to be the most interesting option from an energy recovery point of view. Although this mixture allowed for a slightly lower anaerobic biodegradability (48.2%) than those with higher olive pomace content, it showed the greatest synergistic effect and a significantly lower time to reach 80% of the maximum methane production. Therefore, the proportion 65% pomace–35% pig manure was selected for semi-continuous bench-scale reactors.

### Semi-continuous experiments

#### Mono-digestion of olive pomace

**Fig. 4:**
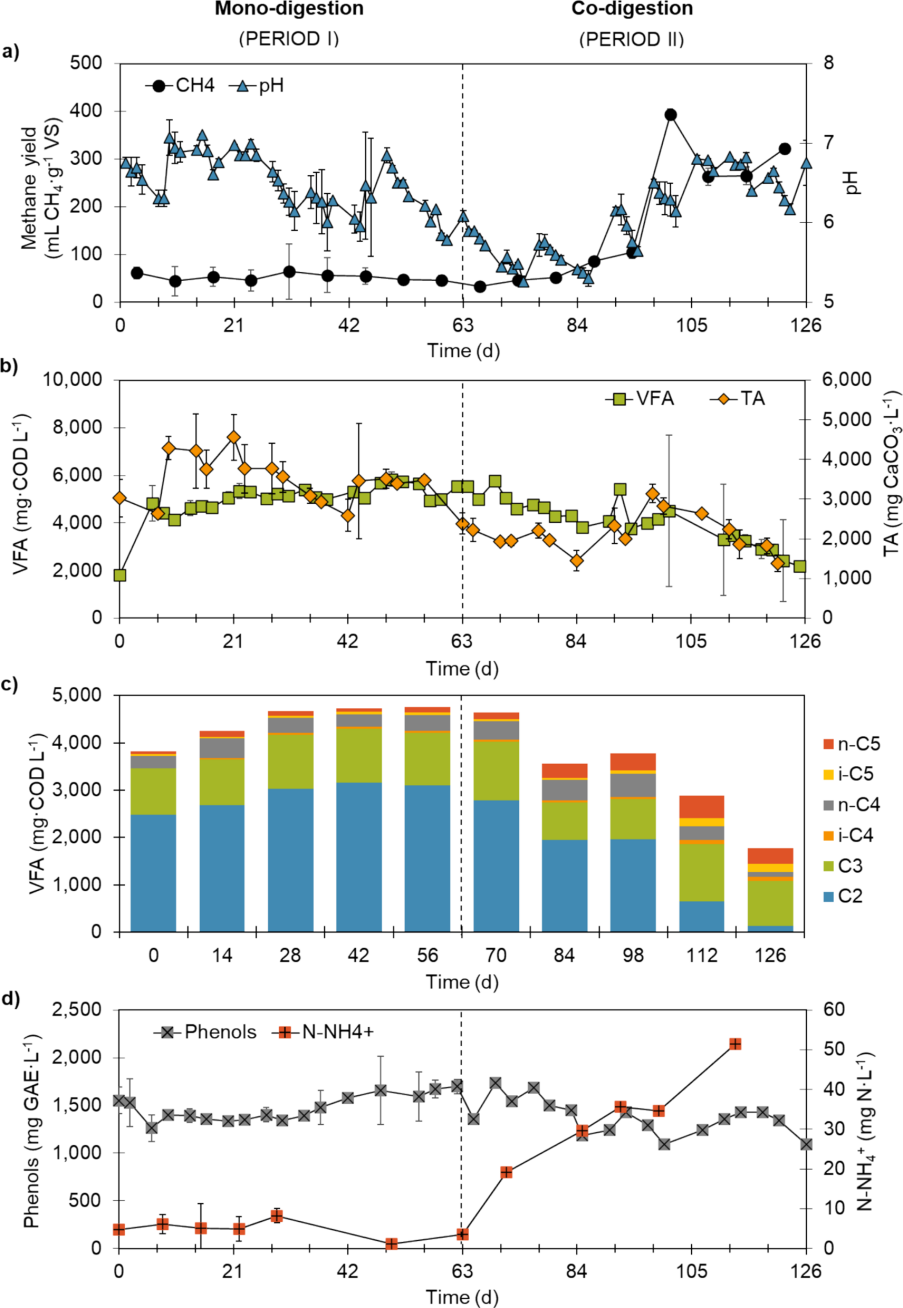
Performance of semi-continuous reactors in terms of methane yield and pH (**a**), volatile fatty acids (VFA) concentration and total alkalinity (TA) (**b**), VFA profile (**c**), phenols and ammonium nitrogen (N–NH_4_^+^) (**d**). Results are expressed as mean and standard deviation (error bars) (*n* = 2). Experiments were run under mesophilic conditions (37 ± 1 °C), with a hydraulic retention time (HRT) of 21 d and an organic loading rate (OLR) of 1 g VS L_R_^−1^ d^−1^. Period I: mono-digestion of two-phase olive pomace; Period II: co-digestion of olive pomace and pig manure (65–35% on a volatile solids (VS) basis). COD: chemical oxygen demand; GAE: gallic acid equivalent

Figure 4a shows the methane yield and pH throughout the operation time of the semi-continuous bench-scale reactors. The mono-digestion of olive pomace (OLR = 1 g VS L_R_^−1^ d^−1^) showed poor performance with a low average methane yield of 52.6 mL CH_4_ g^−1^ VS. The diluted olive pomace fed to the reactors had an acidic pH of 4.58 and negligible alkalinity (Table 1); therefore, it was not possible to maintain the pH within the optimal range for methanogenic archaea. This issue has been reported not only for olive pomace [9], but generally for lignocellulosic materials [37, 38]. Moreover, the substrate was characterised by a high organic content (31 g COD L^−1^), yet the soluble-to-total COD ratio was only 0.39, meaning that most of the organic matter was in particulate form. Thus, the disintegration and hydrolysis of the substrate could also be limiting the process. Moreover, the poor buffering capacity of the reactors, on average 3100 mg CaCO_3_ L^−1^, also prompted the accumulation of VFA, which reached 6 g COD L^−1^ (Fig. 4b). Acetic and propionic acids showed the highest concentration among the measured VFA, which represented 62% and 29%, respectively (Fig. 4c). Similar acid profiles were obtained by Cabrera et al. [39] during pH-controlled fermentation of olive pomace under acidic conditions. In fact, acetic acid concentrations over 3000 mg L^−1^ may indicate that all routes for acetate conversion into methane were compromised during olive pomace mono-digestion [40, 41]. In addition, the concentration of phenolic compounds inside the reactors remained relatively constant, at approximately 1400 mg gallic acid equivalent (GAE) L^−1^, throughout the entire operation time (Fig. 4d). This is near the partial inhibition limit proposed in the literature for anaerobic archaea, i.e., 1500 mg L^−1^, yet far below the inhibitory threshold of 2000 mg L^−1^ reported as inhibiting for fermentative bacteria [36, 42]. The measured concentrations indicate an accumulation rather than degradation of phenolic compounds inside the reactors. Conversely, other authors attributed their removal to the hydrolysation of complex organic matter at high temperatures (180 °C) and the release of phenolic compounds from these matrices [43]. In agreement with this, soluble phenols may be easier to degrade and, therefore, less detrimental for stable anaerobic digestion performance. Besides, the residue used in this study was rich in hydroxityrosol (2750 mg L^−1^) (Table 1). Other inhibitory compounds, such as 3,4-dihydroxyphenylglycol and tyrosol, were also detected in significant concentrations (296 and 799 mg L^−1^, respectively). These compounds have been characterised by their antimicrobial activity, which can be especially detrimental for methanogenic archaea [44]. Nevertheless, reactor inhibition during the mono-digestion period was likely driven by a combination of factors in addition to the presence of phenols, such as an acidic pH and lack of alkalinity.

#### Co-digestion of olive pomace and pig manure

In light of the mono-digestion results, suggesting an inhibition of the methanogenesis, pig manure was added as co-substrate during the second experimental period (days 64–126). During this stage, the anaerobic reactors were fed with olive pomace and pig manure (65% olive pomace and 35% pig manure on a VS basis), keeping the same operational parameters (OLR of 1 g VS L_R_^−1^ d^−1^ and HRT of 21 d). This co-digestion ratio allowed for the dilution of phenolic compounds below inhibition thresholds and increased the buffer capacity to recommended levels [45]. Indeed, the diluted mixture of olive pomace and pig manure had a higher pH (6.12) and alkalinity (~ 1700 mg CaCO_3_ L^−1^) than diluted olive pomace.

Upon steady-state operation (e.g., from day 105 onwards), the average methane yield was 283 mL CH_4_ g^−1^ VS (Fig. 4a), corresponding to over a fivefold increase as compared to the mono-digestion period (Fig. 4a). Indeed, the average anaerobic biodegradability increased from 10% to 35%, while the organic matter removal reached a maximum value of 46% VS. The increase in anaerobic biodegradability and methane yield is likely related to the higher buffer capacity of the digesters, provided by the pig manure used as co-substrate (~ 25.8 g CaCO_3_ L^−1^; Table 1). The pH inside the reactor also increased, reaching values within the optimum range for anaerobic digestion (6.5–7.5) (Fig. 4a). Ammonium levels also increased (Fig. 4d) but remained within the expected range given the characterization of pig manure (Table 1), posing no risk for inhibition [46]. This increment was attributed to the ammonium provided by the pig manure rather than to degradation mechanisms occurring inside the reactors. In addition, the C/N ratio (28.37) was also within the optimum range for anaerobic digestion (20–30) [47]. However, as for the mono-digestion period, the influent showed limited soluble organic matter content (52 g soluble COD kg^−1^) and a soluble-to-total COD ratio of 0.33, meaning that both substrates had most of the organic matter in particulate form; hence, the disintegration and hydrolysis continued to be the limiting steps for the anaerobic co-digestion.

Regarding the VFA concentration, it gradually decreased from 5600 to 2200 mg COD L^−1^ upon co-digestion (Fig. 4b), while the VFA-to-TA ratio decreased from 1.90 to 0.87. According to the literature, when the VFA-to-TA ratio exceeds 0.8 process instability can be expected [48]. However, in this study, a stable performance was observed once the value decreased to approximately 1, which is not uncommon for carbon-rich substrates [49]. Concerning the VFA profile (Fig. 4c), there was a progressive consumption of acetic acid that sharply decreased towards the end of the period (from day 98 onwards). This trend, along with the methane yield increase, confirmed the anaerobic digestion performance recovery with respect to the mono-digestion period. The consumption of acetic acid was accompanied by a relatively high concentration of propionic acid (~ 850 mg COD L^−1^) and longer chain VFA (especially valeric and iso-valeric) in respect to the mono-digestion period. The propionic-to-acetic ratio fluctuated between 1.0 and 6.3. According to the literature, values above 1.4 tend to inhibit acetogenic bacteria, as propionic acid is accumulated and not transformed into acetic acid [50]. However, in this case, it was most probably a consequence of the rapid consumption of acetic acid upon co-digestion, as it decreased from 3520 to 170 mg COD L^−1^, in comparison with the slow conversion rate of propionic acid. Even if the accumulation of propionic acid has been associated with process imbalance and inhibition, it may be considered a consequence rather than a cause of anaerobic digestion failure [51]. For instance, when high concentrations of phenol were added to an anaerobic digester fed with glucose, an increase in propionate resulting from the metabolism of phenol was observed; and despite the high concentration of propionate (2750 mg COD L^−1^), the process was not inhibited and the bioconversion rates remained the same [52]. Therefore, high propionic acid concentration in this study, both during mono- and co-digestion periods, could be explained by phenolic compounds present in olive pomace.

As far as total and soluble phenols are concerned, average concentrations were 1357 and 429 mg GAE L^−1^, respectively. Therefore, when comparing the mono-digestion and co-digestion periods, no relevant differences were found between the concentration of phenolic compounds in the effluent. The recovery of the reactors upon co-digestion may not only be related to the dilution of phenolic compounds in the influent, but also to the characteristics of pig manure (i.e., higher pH, alkalinity and nitrogen content than olive pomace) which balanced the substrate composition. Overall, the co-digestion of olive pomace with pig manure improved the anaerobic digestion performance, process stability and methane yield.

Compared to the BMP tests, semi-continuous reactors provided key additional insights. Mono-digestion of olive pomace performed well in batch assays, likely due to the role of the inoculum (digested sludge), which balanced the C/N ratio, provided alkalinity and buffering capacity, maintained a neutral pH favourable to methanogenic archaea, diluted inhibiting compounds like phenols, and supplied trace elements lacking in olive pomace. However, without this masking effect attributed to the inoculum, semi-continuous operation evidenced the deficiencies of olive pomace that constrain its mono-digestion. By contrast, co-digestion with pig manure enhanced the conditions for stable operation and sustainable methane production over time. Despite the relatively high concentration of phenolic compounds, it was possible to maintain stable reactor performance [53]. Furthermore, the use of an adapted inoculum resulted in higher methane yields, likely due to the presence of microbial communities with increased resistance to inhibitory compounds, such as phenols, and enriched populations of specialised hydrolytic bacteria. These findings emphasise the importance of semi-continuous reactors for evaluating long-term process performance.

#### Microbial population dynamics

**Fig. 5:**
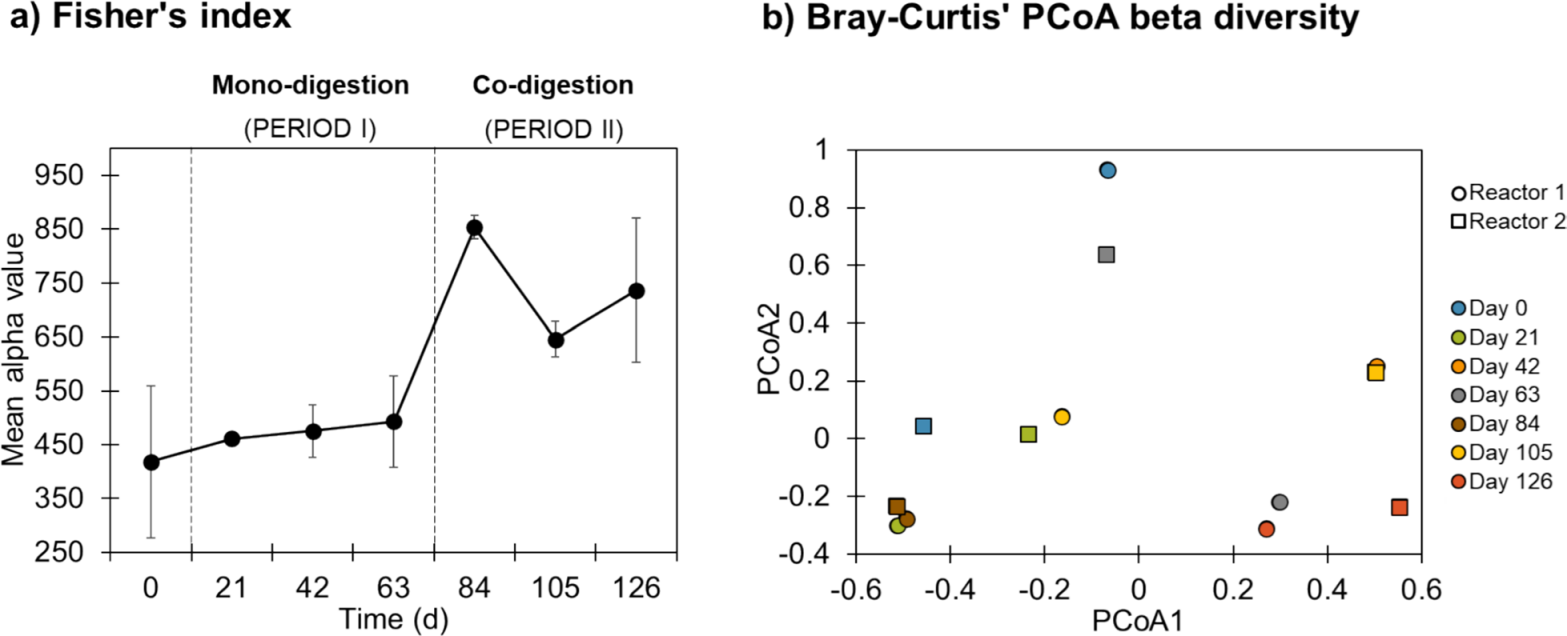
Alpha diversity (**a**) and beta diversity (**b**) analysis during the mono-digestion (days 1–63) and co-digestion (days 64–126) of olive pomace and pig manure in semi-continuous anaerobic reactors. Day 0 corresponds to the sample of the inoculum. **a** Results are expressed as mean and standard deviation (error bars) (*n* = 2). **b** Principal coordinates analysis (PCoA) plot generated using Bray–Curtis dissimilarity, showing the ordination of samples according to differences in microbial community composition

A metagenomic study was conducted upon the transition of semi-continuous anaerobic reactors from mono-digestion to co-digestion of olive pomace and pig manure to observe the evolution of the microbial community over time and its response to changes in substrate composition. In this way, the alpha diversity index and beta diversity were determined over the whole experimental period (Fig. 5). As shown in Fig. 5a, the diversity remained relatively constant during the mono-digestion period (from days 1 to 63), indicating that the community was well-adapted to fermenting olive pomace. However, by adding pig manure (from day 64 onwards), the diversity increased and process performance improved (as described in Sect. "Co-digestion of olive pomace and pig manure"). The greater ecological niche heterogeneity and the expansion of the microbial community were likely due to the nutrients supply, pH, buffer capacity and microbiome present in the pig manure added as co-substrate.

The beta diversity analysis using the Bray–Curtis PCoA plot (Fig. 5b) showed a clear difference between the mono- and co-digestion of olive pomace with pig manure. Indeed, the microbial community at day 63 (mono-digestion) clusters separately from co-digestion points, confirming that co-digestion fundamentally altered the community structure. Differences among point positions suggest that the microbial communities were more homogeneous during the mono-digestion period (days 0, 21, 42 and 63), while they diversified upon co-digestion (days 84, 105 and 126), likely due to the coexistence of new micro-organisms. This confirms that microbial community variability is driven not only by changes in relative abundance but also by the establishment of new functional groups. The increased microbial diversity upon co-digestion suggests improved ecosystem resilience and metabolic versatility [54]. Separation between replicates may reflect temporal dynamics and inherent stochastic variation between reactors due to small differences in microbial growth, environmental conditions, or reactor-specific microenvironments (Table S1).

**Fig. 6:**
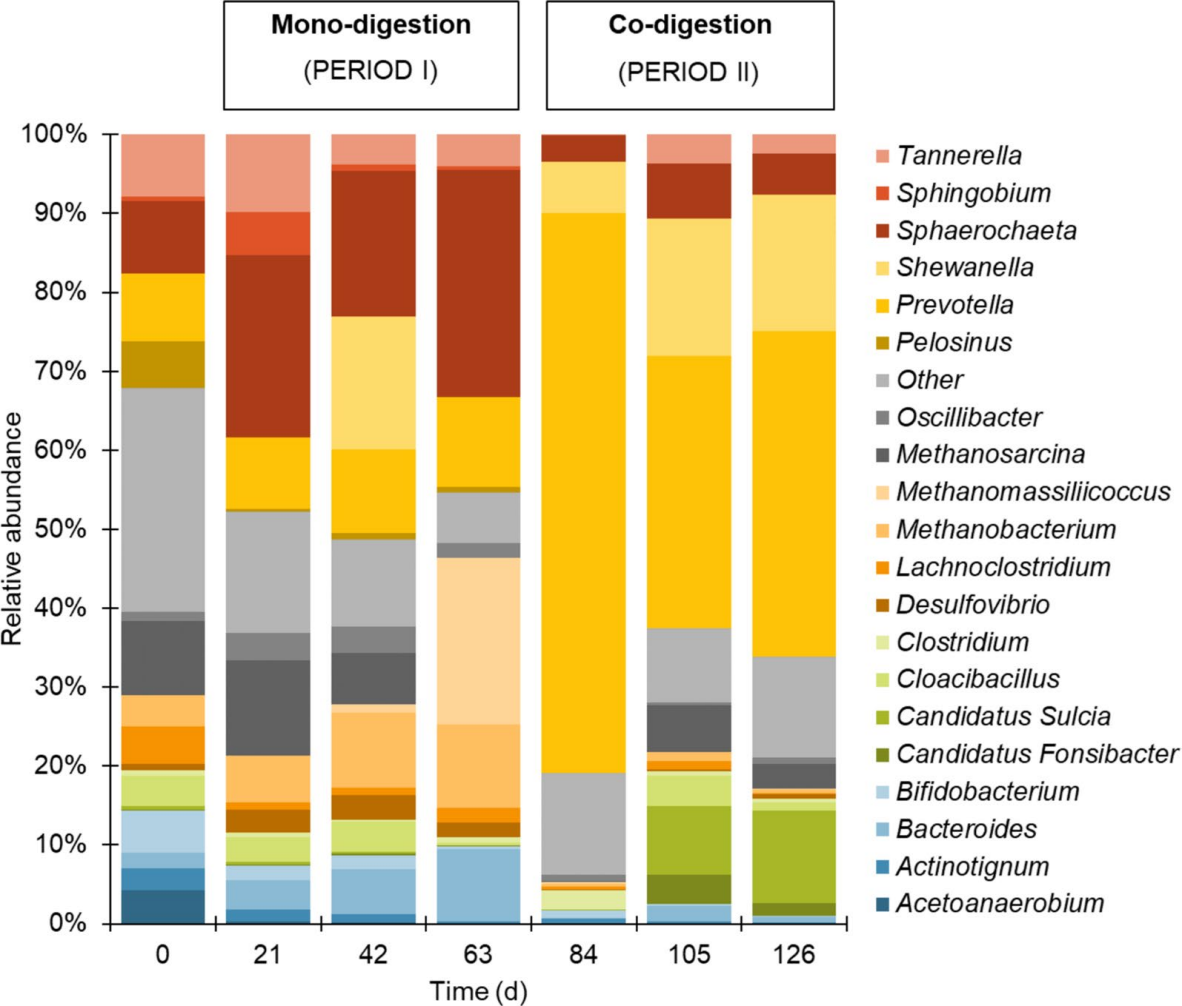
Relative abundance of the top 20 species present in semi-continuous anaerobic reactors upon mono-digestion (days 1–63) and co-digestion (days 64–126) of olive pomace and pig manure. Day 0 corresponds to the sample of the inoculum. Results represent mean values of replicates from two reactors

During the mono-digestion period, significant shifts in microbial composition were observed (Fig. 6). *Bacteroides*, *Clostridium*, and *Prevotella*, recognised for their ability to degrade complex polysaccharides, steadily declined over time [55]. This decrease indicates that their ecological roles were gradually outcompensated by more specialised fermentative species, particularly *Sphaerochaeta*, which efficiently ferments hexose and pentose sugars, the monomers of hemicellulose [56]. *Sphaerochaeta* progressively became the dominant genus by day 63, exceeding 30% relative abundance. This strictly anaerobic genus converts hexoses and pentoses into ethanol, acetate, and formate as major end products and can also produce lactic acid, hydrogen, and carbon dioxide during carbohydrate fermentation [57, 58]. The dominance of this genus at the end of the mono-digestion phase is in accordance with the low pH values and high content of VFA observed at the end of the mono-digestion phase, reflecting active fermentative metabolism.

However, with the addition of pig manure and the consequent improvement of the digestion process, the relative abundance of this genus decreased concomitantly with the increase of *Prevotella*. *Prevotella* was only present at low levels during the mono-digestion period, but raised sharply upon co-digestion, reaching 73.8% relative abundance at day 84. *Prevotella* is one of the most predominant genera in the pig intestine and is well-adapted to the degradation of lignocellulosic substrates in both the rumen and the gut. The addition of pig manure, which harbours a high abundance of *Prevotella*, to olive pomace with a high lignin content likely promoted the dominance of this genus, enhancing the breakdown of complex polysaccharides and improving the fermentation efficiency [59–62].

Regarding the Archaea domain, the relative abundance of *Methanobacterium*, and *Methanomassiliicoccus* initially increased, but dropped significantly after day 63, due to changes in syntrophic interactions and/or the addition of pig manure as co-substrate. The decrease in the abundance of methanogens such as *Methanosarcina* from day 0 to day 63 is in accordance with the low pH and high content of VFA at the end of the mono-digestion period. *Methanosarcina* re-emerged after day 84, potentially indicating shifts in methanogenesis pathways from hydrogenotrophic to acetoclastic, which is indicative of increased stability of the anaerobic digestion process [63]. Indeed, the recovery of the anaerobic digestion process upon co-digestion can be partially explained by the increase in *Methanosarcina* abundance.

### Energy assessment of a full-scale co-digestion plant

**Fig. 7:**
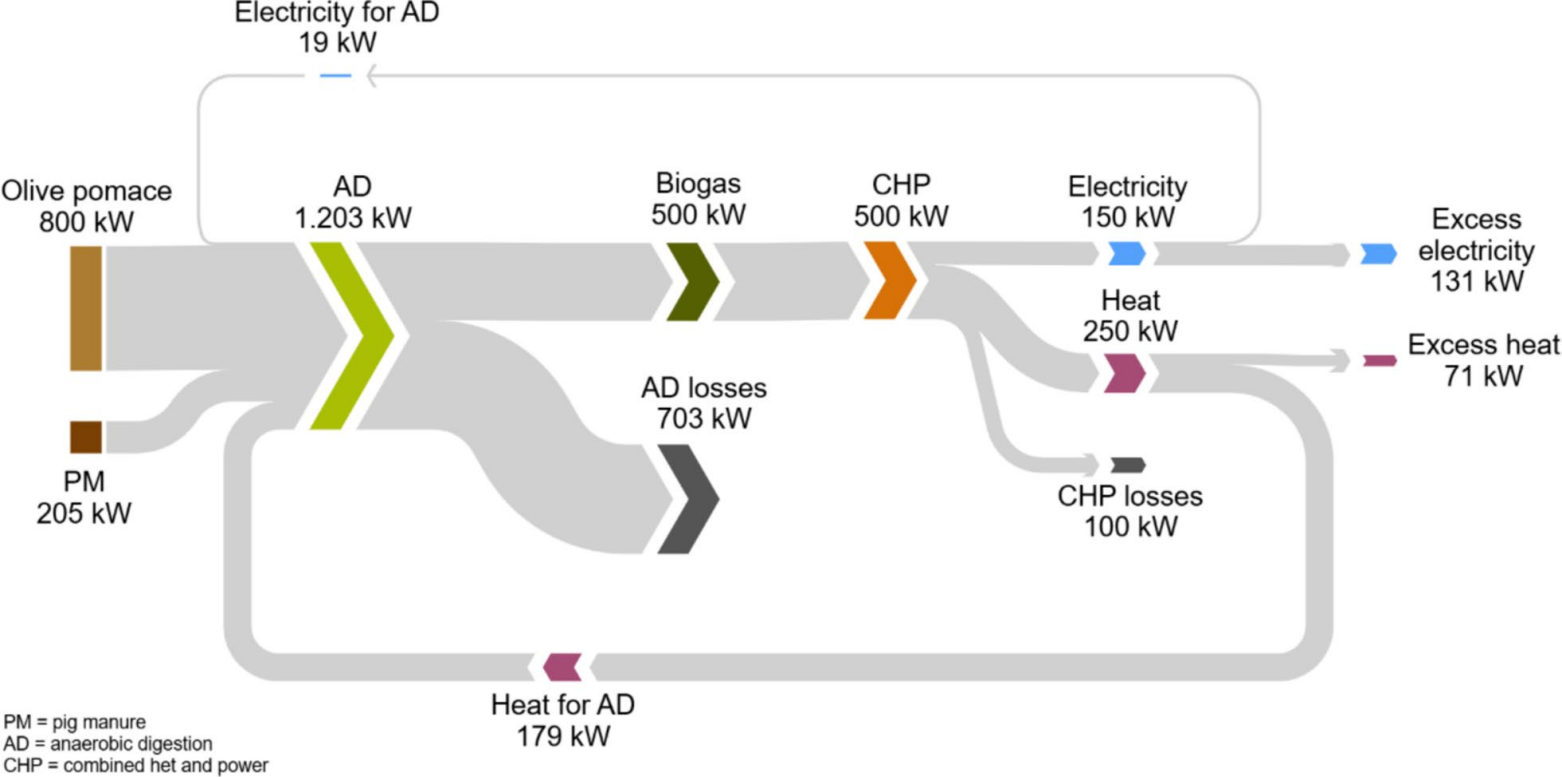
Sankey diagram showing the energy flows within the anaerobic digestion—combined heat and power (CHP) facility. The plant was designed to treat 4000 t y^−1^ of olive pomace and 2200 t y^−1^ of pig manure; nominal capacity of the CHP unit is 150 kW_e_ (250 kW_th_), operating 8000 h y^−1^. Results are based on experimental methane yields from semi-continuous reactors obtained in this study

Experimental data from the co-digestion stability period was used to perform the energy balance for a full-scale facility integrating an anaerobic digester and a biogas-fed CHP unit (Fig. 1). The energy flows for the proposed facility are shown in Fig. 7. Based on experimental results, it was estimated that the proposed digester would produce 1233 Nm^3^ CH_4_ d^−1^ to be fed to the CHP unit. Energy balances showed that the CHP plant would be able to provide enough thermal and electric energy for the facility to be self-sufficient. The CHP unit would have an electric rated power output of 150 kW_e_ and a useful thermal energy output of 250 kW_th_. Approximately, 12% of the electricity produced (152 MWh y^−1^) would be employed to cover the electricity needs of the facility (i.e., pumping and stirring). Therefore, excess electricity (1074 MWh y^−1^) could be sold to the grid. Thermal energy produced (2043 MWh y^−1^) would be mostly utilised to cover the heat demand of the biogas plant (1466 MWh y^−1^), i.e., maintaining mesophilic conditions in the reactor by both raising the temperature of the co-substrates and compensating for thermal losses. Excess thermal energy was estimated as 28% of the CHP output (577 MWh y^−1^), and could be used to cover the heat demand of the olive mill or else other nearby industries. Energy losses in the anaerobic digestion process (i.e., 58%) were estimated as the difference between the energy content of the substrates, calculated from their theoretical heating values [64, 65], and the energy recovered as methane through anaerobic digestion. These losses were attributed to three main factors. First, thermal energy dissipated through the digester walls. Second, the presence of non-biodegraded (recalcitrant) organic matter in the digestate. Third, olive pits present in the olive pomace that cannot be transformed into biogas by anaerobic micro-organisms. Consequently, it is recommended to separate this fraction for alternative valorisation pathways such as pyrolysis.

Beyond energy valorisation, future research should explore the upstream recovery of high-value compounds such as lignin and phenols [66, 67] prior to the anaerobic digestion, aiming to transform biogas plants into integrated biorefineries capable of generating multiple products from residual biomass sources.

## Conclusions

This study evaluated the co-digestion of olive pomace with pig manure, given their complementary composition. BMP tests confirmed the potential of olive pomace for biogas production and revealed synergistic effects upon co-digestion with pig manure. However, under semi-continuous operation, the mono-digestion of olive pomace failed, which was attributed to the acidic pH, lack of alkalinity and presence of inhibitory compounds (phenols). Nevertheless, co-digestion with pig manure enabled a stable process performance, in agreement with previous studies on nitrogen-rich feedstocks. Indeed, co-digestion increased the methane yield by fivefold as compared to the mono-digestion (283 *vs* 53 mL CH_4_ g^−1^ VS) and enhanced microbial diversity, ecosystem resilience and metabolic versatility. Specifically, the relative abundance of *Prevotella*, *Lentimicrobium* and *Methanosarcina* increased, while *Sphaerochaeta* decreased upon co-digestion. An energy assessment of a full-scale plant indicated that the process would be energetically self-sufficient, and even generate surplus energy. Future work should focus on pilot-scale validation and addressing limitations such as olive pomace seasonality. This would facilitate technology implementation and contribute to the sustainability of the agro-industrial sector and the energy transition goals.

## Supplementary Information

Additional file 1 (DOCX 18 KB)

## Data Availability

The data sets used and/or analysed during the current study are available from the corresponding author on reasonable request.
