# Supplementary material for: Enhancing methane yield and microbial resilience in olive pomace anaerobic digestion via co-digestion with pig manure
Source: Biotechnol Biofuels Bioprod. 2025 Nov 18;18:114. doi: 10.1186/s13068-025-02711-9 (PMC12625033; doi:10.1186/s13068-025-02711-9)
Supplement: Supplementary file 1 — Additional file 1 (DOCX 18 KB) [file 13068_2025_2711_MOESM1_ESM.docx]

**Enhancing methane yield and microbial resilience in olive pomace anaerobic digestion via co-digestion with pig manure**

**Supplementary material**

**Table S1.** Bray–Curtis dissimilarity matrix showing pairwise differences in microbial community composition among samples. Values range from 0 (identical communities) to 1 (completely dissimilar communities).

|  | **R1 day 0** | **R2 day 0** | **R1 day 21** | **R2 day 21** | **R1 day 42** | **R2 day 42** | **R1 day 63** | **R2 day 63** | **R1 day 84** | **R2 day 84** | **R1 day 105** | **R2 day 105** | **R1 day 126** | **R2 day 126** |
| --- | --- | --- | --- | --- | --- | --- | --- | --- | --- | --- | --- | --- | --- | --- |
| **R1 day 0** | 0 | 0.513 | 0.59 | 0.811 | 0.902 | 0.896 | 0.834 | 0.64 | 0.716 | 0.675 | 0.855 | 0.911 | 0.852 | 0.912 |
| **R2 day 0** | 0.513 | 0 | 0.243 | 0.557 | 0.766 | 0.694 | 0.643 | 0.659 | 0.332 | 0.301 | 0.629 | 0.777 | 0.623 | 0.743 |
| **R1 day 21** | 0.59 | 0.243 | 0 | 0.421 | 0.752 | 0.56 | 0.63 | 0.64 | 0.245 | 0.283 | 0.479 | 0.726 | 0.588 | 0.662 |
| **R2 day 21** | 0.811 | 0.557 | 0.421 | 0 | 0.797 | 0.544 | 0.745 | 0.797 | 0.492 | 0.48 | 0.241 | 0.773 | 0.708 | 0.735 |
| **R1 day 42** | 0.902 | 0.766 | 0.752 | 0.797 | 0 | 0.427 | 0.641 | 0.863 | 0.755 | 0.757 | 0.812 | 0.084 | 0.638 | 0.437 |
| **R2 day 42** | 0.896 | 0.694 | 0.56 | 0.544 | 0.427 | 0 | 0.432 | 0.757 | 0.616 | 0.654 | 0.561 | 0.427 | 0.439 | 0.275 |
| **R1 day 63** | 0.834 | 0.643 | 0.63 | 0.745 | 0.641 | 0.432 | 0 | 0.678 | 0.622 | 0.605 | 0.764 | 0.658 | 0.258 | 0.375 |
| **R2 day 63** | 0.64 | 0.659 | 0.64 | 0.797 | 0.863 | 0.757 | 0.678 | 0 | 0.561 | 0.563 | 0.815 | 0.866 | 0.729 | 0.831 |
| **R1 day 84** | 0.716 | 0.332 | 0.245 | 0.492 | 0.755 | 0.616 | 0.622 | 0.561 | 0 | 0.177 | 0.513 | 0.745 | 0.595 | 0.698 |
| **R2 day 84** | 0.675 | 0.301 | 0.283 | 0.48 | 0.757 | 0.654 | 0.605 | 0.563 | 0.177 | 0 | 0.533 | 0.767 | 0.574 | 0.715 |
| **R1 day 105** | 0.855 | 0.629 | 0.479 | 0.241 | 0.812 | 0.561 | 0.764 | 0.815 | 0.513 | 0.533 | 0 | 0.785 | 0.731 | 0.742 |
| **R2 day 105** | 0.911 | 0.777 | 0.726 | 0.773 | 0.084 | 0.427 | 0.658 | 0.866 | 0.745 | 0.767 | 0.785 | 0 | 0.647 | 0.436 |
| **R1 day 126** | 0.852 | 0.623 | 0.588 | 0.708 | 0.638 | 0.439 | 0.258 | 0.729 | 0.595 | 0.574 | 0.731 | 0.647 | 0 | 0.393 |
| **R2 day 126** | 0.912 | 0.743 | 0.662 | 0.735 | 0.437 | 0.275 | 0.375 | 0.831 | 0.698 | 0.715 | 0.742 | 0.436 | 0.393 | 0 |
